# Botulinum Toxin Combined with Robot-Assisted Therapy for Post-Stroke Spasticity: A Systematic Review

**DOI:** 10.3390/toxins17120569

**Published:** 2025-11-25

**Authors:** Salvatore Facciorusso, Stefania Spina, Mirko Filippetti, Rajiv Reebye, Gerard E. Francisco, Andrea Santamato

**Affiliations:** 1 Spasticity and Movement Disorders “ReSTaRt” Unit, Physical Medicine and Rehabilitation Section, Azienda Ospedaliero-Universitaria Ospedali Riuniti Di Foggia, 71122 Foggia, Italy; s.facciorusso89@gmail.com (S.F.);; 2 Department of Medical and Surgical Science, University of Foggia, 71122 Foggia, Italy; 3 Department of Physical Medicine and Rehabilitation, Department of Neurosciences, Biomedicine and Movement Sciences, University of Verona, 37129 Verona, Italy; 4 Division of Physical Medicine and Rehabilitation, Faculty of Medicine, University of British Columbia, Vancouver, BC V5Z 2G9, Canada; 5 Department of Physical Medicine & Rehabilitation, University of Texas Health McGovern Medical School, Houston, TX 77030, USA

**Keywords:** stroke, spasticity, botulinum toxin, robot-assisted therapy

## Abstract

(1) Background: Post-stroke spasticity limits motor recovery and independence. Combining botulinum toxin type-A (BoNT-A) injection with intensive, task-specific robot-assisted therapy (RAT) might enhance neuroplasticity and functional gains, but its additive effect and optimal timing are uncertain. (2) Methods: We systematically searched major medical databases and trial registries up to April 2025 for randomized controlled trials in adults with post-stroke spasticity comparing botulinum toxin type-A injection plus RAT with toxin injection plus conventional therapy, or RAT alone with RAT combined with toxin injection. Risk of bias was assessed using the RoB 2 tool, and findings were synthesized narratively. (3) Results: Seven trials (*n* = 229) were included. Across all studies, toxin treatment reduced spasticity within groups, whereas additional spasticity reduction with RAT versus conventional rehabilitation was inconsistent. In contrast, several lower-limb trials reported greater improvements in walking capacity and balance when RAT was added, while upper-limb trials showed comparable motor recovery across treatment arms with occasional advantages in strength and movement quality. A pilot four-arm study suggested that starting RAT around four weeks after injection may maximize upper-limb motor gains. (4) Conclusions: The combination of BoNT-A with RAT appears safe and is particularly promising for gait rehabilitation, but further research is needed to define optimal timing and protocols.

## 1. Introduction

Stroke is a leading cause of death and long-term disability worldwide and leaves many of the estimated 94 million survivors with persistent neurological deficits that compromise motor function, independence in daily activities, and quality of life [1]. Spasticity, defined as a velocity-dependent increase in muscle tone with exaggerated stretch reflexes [2], affects up to 40% of stroke survivors and could be a major barrier to effective rehabilitation [3]. Over recent decades, management of post-stroke spasticity has evolved substantially [4]. Botulinum toxin type-A (BoNT-A) is the first-line treatment for focal spasticity, with robust evidence that it reduces muscle tone and improves passive function [5], although its isolated effect on active motor performance—particularly in chronic stroke—remains less clear [5].

Alongside pharmacologic approaches, technological innovations have introduced robot-assisted therapy (RAT) as a way to deliver high-intensity, repetitive, task-specific training with precise control and objective feedback [6]. Robot-assisted devices can augment conventional rehabilitation and have shown favorable effects on motor recovery, especially in patients with moderate-to-severe impairment [7].

Recent years have seen a growing interest in combining toxin treatment with intensive rehabilitation approaches, including RAT [8]. Toxin treatment reduces muscle overactivity and may create a window of opportunity for more effective motor training [9,10], while RAT provides consistent, intensive practice of movement patterns. However, the optimal protocols, timing, and comparative effectiveness of this combined approach remain uncertain.

Previous systematic reviews have addressed BoNT-A for spasticity [5,11,12], adjunct therapies after BoNT-A [13,14], or RAT as a standalone intervention [6,7], but none have specifically synthesized randomized trials of BoNT-A combined with RAT.

This systematic review aimed to address this specific gap by rigorously evaluating the efficacy of combining robot-assisted therapy with BoNT-A injections compared to other relevant treatment approaches (e.g., BoNT-A plus conventional therapy, robotic therapy alone, BoNT-A alone, or standard care) in patients with post-stroke spasticity. We focus on spasticity reduction as the primary outcome and on motor and functional outcomes as secondary endpoints, including the influence of upper- versus lower-limb application and the timing of RAT relative to toxin injection.

## 2. Results

### 2.1. Study Selection

**Figure 1:**
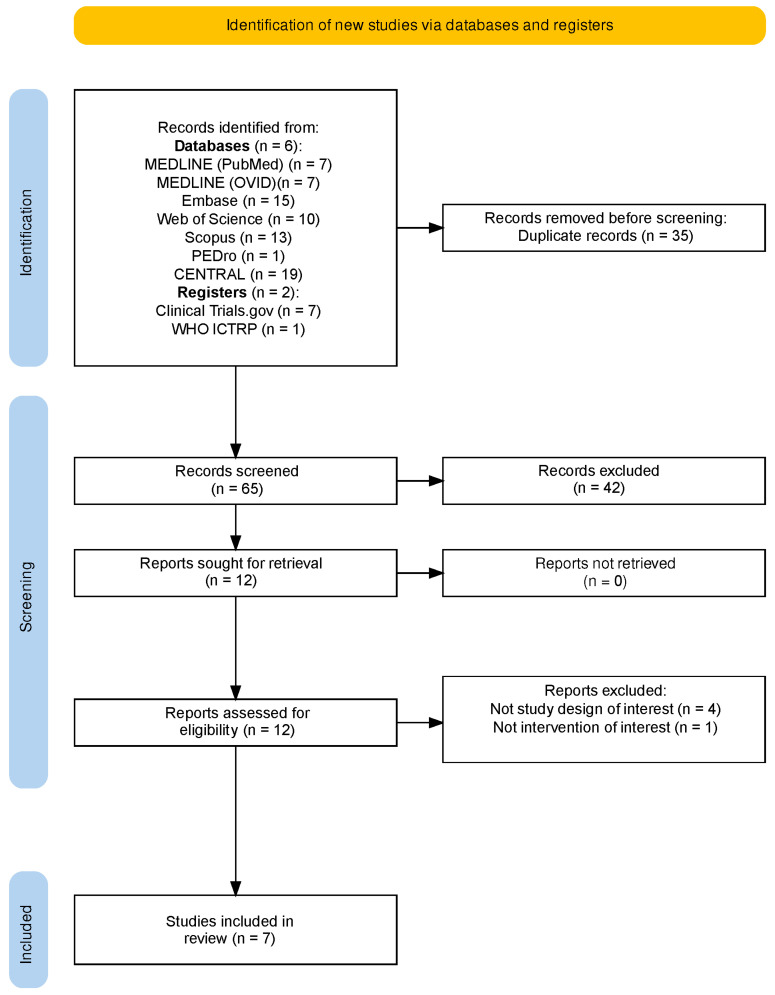
PRISMA flow chart.

The initial database search identified 80 records, spanning from database inception to April 2025. After removing duplicates and screening titles and abstracts, seven randomized controlled trials met the inclusion criteria; most exclusions were due to non-randomized design, lack of a RAT component, or absence of toxin injection (Figure 1 shows the PRISMA flowchart).

### 2.2. Study Characteristics

The seven chronic-stroke RCTs (*n* = 229) were published between 2015 and 2025 and included four upper-limb [15,16,17,18] and three lower-limb [19,20,21] trials, with small sample sizes (15–48 participants) and mean ages from 44 to 65 years.

### 2.3. Participant Characteristics

Across trials, participants had clinically significant upper- or lower-limb spasticity of at least mild-to-moderate severity and residual motor function compatible with training, such as minimal upper-limb movement or the ability to walk short distances.

Table 1 summarizes further details of the main characteristics of the studies included.

### 2.4. Interventions Characteristics

Across all studies, at least one treatment arm combined toxin injection with a subsequent rehabilitation program, allowing comparisons with other active rehabilitation strategies with or without toxin injection.

#### 2.4.1. BoNT-A Administration

The target muscles were selected based on the study’s focus, primarily involving upper limb spastic muscles such as elbow flexors, forearm pronators, and wrist/finger flexors [15,16,17,18], or lower limb muscles, particularly the triceps surae for spastic equinus foot [19,20,21]. Doses were individualized according to spasticity severity and distribution, sometimes with an upper dose limit, and injections were generally guided by ultrasound [15,16,17,20] or electrical stimulation [18,19,21] to improve accuracy.

#### 2.4.2. Rehabilitation Protocols

The timing of rehabilitation onset after toxin injection varied from the same day [20] to approximately four weeks later [16,17,21], with one four-arm trial designed specifically to test different sequencing schedules [15]. Experimental groups received intensive robot-assisted training of the injected limb—typically 2–5 sessions per week for 2–8 weeks, with 30–90 min per session—using either gait-training systems or upper-limb robots. Comparator groups most often received toxin injection plus conventional physiotherapy or occupational therapy (stretching, strengthening and task-oriented exercises); other comparators included toxin injection plus electrical stimulation or mirror therapy, or RAT alone. One trial without toxin in the control arm allowed direct assessment of the added value of toxin injection over robotic training alone. Table 2 shows further details on Rehabilitation protocols.

### 2.5. Risk of Bias Assessment

All trials were judged to have an overall “some concerns” risk of bias, mainly due to potential selective reporting. Adherence to intended interventions and outcome measurement was generally adequate, while randomization procedures and handling of missing data were less consistently reported. The findings should therefore be interpreted cautiously and viewed as preliminary.

**Figure 2:**
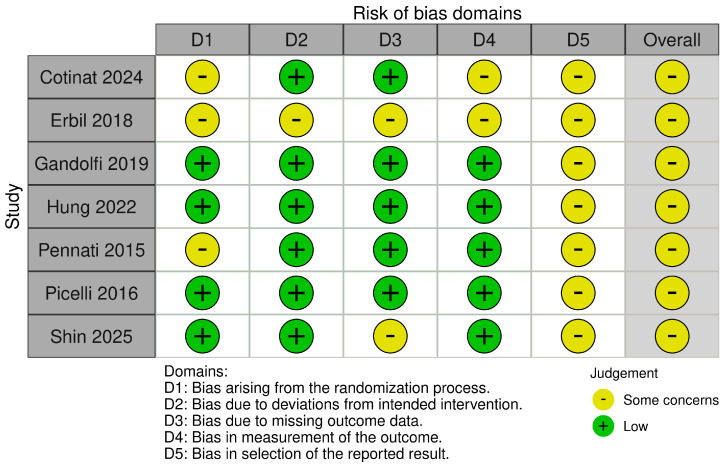
Risk of bias (ROB 2) summary [15,16,17,18,19,20,21].

**Figure 3:**
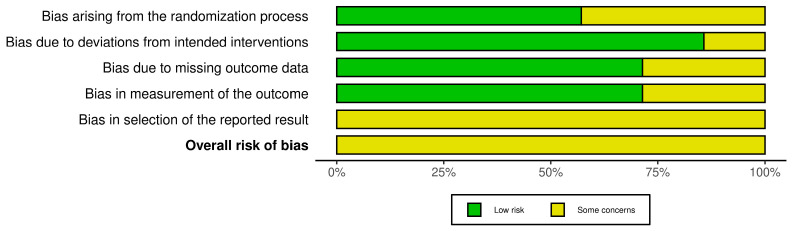
Risk of bias (ROB 2) graph.

Risk of bias summary and graph are summarized in Figure 2 and Figure 3.

### 2.6. Effects of Interventions

Statistical pooling of results through meta-analysis was not feasible due to substantial clinical and methodological heterogeneity across studies. Key factors preventing meta-analysis included: (1) varying robot types and different rehabilitation protocols; (2) different BoNT-A formulations and dosing protocols, with doses ranging based on individualized protocols; (3) diverse timing and frequency of intervention.

A narrative synthesis was conducted to summarize and explain the findings of included studies. Table 2 highlights details about the primary and secondary outcomes.

#### 2.6.1. Primary Outcomes: MAS

All seven studies assessed spasticity, primarily using the MAS. Across studies, groups receiving toxin injection showed consistent within-group reductions in spasticity from baseline to post-treatment. Between-group comparisons were mixed: in most trials, toxin injection plus RAT achieved similar spasticity reductions to toxin injection plus conventional therapy or plus electrical stimulation for both upper [16,17] and lower limbs [19,21], whereas a few studies reported larger MAS improvements when toxin injection was combined with robotic training compared with robotic training alone or with no toxin injection [15,18]. Overall, the combined approach did not reliably enhance antispastic effects beyond those of toxin treatment itself.

#### 2.6.2. Motor Function

Among upper-limb trials, motor recovery was mainly evaluated with the Fugl–Meyer Assessment. Across studies, toxin injection plus RAT and toxin injection plus conventional task-oriented training generally produced similar FMA gains [17], although robotic training was associated with greater improvements in muscle strength and more favorable agonist activation patterns in some studies [16]. One small study observed numerically larger FMA gains with robotic training alone than with the combined approach [18], highlighting that the added value of toxin injection for upper-limb motor recovery remains uncertain. In Shin et al. [15], no short-term differences emerged in the first four weeks, but the group that started robotic training about four weeks after injection showed the largest eight-week improvements in motor scores and kinematic parameters.

For the lower limb, findings were more consistent. All three gait-focused trials reported that adding RAT to toxin injection led to greater improvements in walking capacity, gait speed and balance than toxin injection plus conventional physiotherapy [19,21] or plus electrical stimulation alone [20]. Measures of walking distance, gait speed and balance improved in both groups, but gains were typically larger when robotic gait training was included.

#### 2.6.3. Functional Outcomes, Daily Activities, and Quality of Life

Several measures have been used to assess the impact of the interventions on daily function and quality of life, with varied results.

Across studies, toxin injection combined with any active rehabilitation—robot-assisted, conventional, or mirror therapy—produced modest improvements in self-reported arm use and functional independence [17,18], with no clear or consistent advantage of the robotic approach. In several trials, changes in quality of life and broader stroke-impact scores were small and not statistically significant [15], and accelerometer-based measures of real-world arm activity showed minimal change [17].

#### 2.6.4. Adverse Events

No serious adverse events directly attributable to the combined interventions were reported across the studies. Minor issues related to the robotic devices, such as transient skin irritation or musculoskeletal discomfort [21], occurred infrequently and rarely led to treatment discontinuation [19].

### 2.7. Synthesis of Results

The evidence suggests that while adding robotic therapy to BoNT-A treatment does not provide additional benefit for spasticity reduction over conventional therapy, it appears to yield greater to other active therapies, but may be optimized when robotic therapy is initiated approximately four weeks after injection.

## 3. Discussion

This systematic review synthesized seven randomized trials evaluating the combination of toxin injections and RAT for post-stroke spasticity.

In line with our prespecified outcome hierarchy, we first considered spasticity and then examined motor, gait and broader functional outcomes.

Five of seven studies showed equivalent spasticity reduction outcomes when comparing BoNT-A plus RAT versus BoNT-A plus conventional therapy, as measured by MAS score [16,17,19,20,21]. This pattern held across both upper limb studies and lower limb investigations, suggesting that the type of adjunct therapy, whether robotic or conventional, may be less critical for spasticity reduction than the toxin injection itself. However, two studies provided evidence for enhanced antispastic effects with specific combined approaches [15,18].

In contrast, a broadly consistent pattern emerged for functional outcomes, with all three lower-limb studies reporting superior walking-related improvements with robot-assisted gait training [19,20,21]. However, these conclusions are based on only three small gait-focused trials and should therefore be interpreted cautiously. For the upper limb, RAT produced motor recovery gains comparable to other active interventions like conventional or mirror therapy [17]. However, some evidence suggested that the robotic approach yielded superior improvements in underlying physiological measures, such as muscle strength and more effective agonist activation patterns [16], especially when robotic training is scheduled during the post-injection period around four weeks after injection [15]. Despite these clinical gains, a significant challenge remains, as objective measurements revealed minimal translation of improvements into spontaneous, real-world arm use.

The mechanisms underlying these differential outcomes likely involve activity-dependent neuroplasticity. The combination of BoNT-A and RAT appears to facilitate cortical reorganization through complementary mechanisms [22,23]. BoNT-A achieves a targeted reduction in muscle overactivity, diminishing pathological activation patterns that compete with voluntary muscle contractions [24]. During this critical period of attenuated spasticity, RAT delivers the high-intensity, repetitive practice necessary to drive neuroplastic changes in the motor cortex and corticospinal tract [10,25].

Electromyographic evidence from the included studies substantiates this mechanistic framework [16]. This evidence shows improved muscle activation patterns and reduced co-contraction in patients who received the combined therapy. These neurophysiological changes suggest a benefit that extends beyond simple tone reduction to a more fundamental reorganization of motor control strategies [26]. Furthermore, the documented improvements in muscle strength and agonist activation support the idea that the therapy helps restore voluntary motor control, rather than merely providing a passive reduction in tone [16].

The temporal relationship between BoNT-A administration and robot-assisted therapy initiation has emerged as a critical determinant of therapeutic efficacy. The timing findings from the four-arm upper-limb trial are particularly informative [15]. In that study, the schedule in which robotic training started approximately four weeks after toxin injection yielded the largest improvements in motor scores and kinematic measures over eight weeks, despite similar short-term changes in spasticity [15]. Taken together with the known pharmacodynamics of BoNT-A these data support the hypothesis that scheduling robot-assisted training around week four may maximize the opportunity to consolidate more selective and less spastic movement patterns [27].

Our results complement earlier reviews of BoNT-A and adjunctive rehabilitation. Prior syntheses reported that pairing toxin injections with intensive therapies can enhance motor outcomes compared with toxin alone, although evidence quality was limited and robot-assisted interventions were rarely analyzed separately [11]. Meta-analyses of RAT itself show improvements in activities of daily living and limb function after stroke, particularly in more severely impaired patients [6].

Our findings extend this evidence by demonstrating that combining RAT with BoNT-A might provide additional functional benefits beyond either intervention alone, particularly for lower limb rehabilitation.

Translating these findings into routine practice is constrained by the considerable heterogeneity and resource requirements of robotic systems. The included trials used diverse gait trainers and upper-limb devices, which differ in degrees of freedom, assistance modes and training paradigms; these differences likely influence both dose and quality of practice and complicate cross-study comparisons [28]. In addition, RAT requires substantial capital investment, dedicated space and specialized staff, raising questions about cost-effectiveness and equitable access, particularly in resource-limited settings [6]. Given that toxin injections themselves are already costly and need to be repeated, health-economic evaluations of combined programs are a priority. Cost-effective service delivery models, including group-based sessions, hybrid approaches, or technology-assisted home programs, warrant exploration to enhance accessibility [29,30].

Implementation strategies should focus on identifying patient subgroups that are most likely to benefit from combined therapy. Simple clinical predictors such as baseline spasticity severity, residual voluntary movement and walking ability are promising starting points for tailoring treatment and for designing future pragmatic trials [31,32]. More advanced predictive biomarkers, including neuroimaging measures, diffusion tensor imaging of corticospinal tract integrity, and transcranial magnetic stimulation, may enable personalized treatment selection.

Future research should primarily address three questions. First, adequately powered, multicentre randomized trials using standardized outcome sets are needed to clarify the magnitude and durability of functional benefits of combined toxin injection and RAT, including follow-up beyond six to twelve months. Second, timing of robotic training relative to injection should be systematically varied—particularly around the two- to six-week post-injection window identified in this review—to define the most effective scheduling. Third, cost-effective delivery models are required, such as targeted use of robotic gait training for those with severe gait limitations, group-based or hybrid programs, and integration with home-based technologies, ideally informed by simple clinical predictors such as baseline spasticity severity, residual voluntary movement and patient-reported measures [33,34].

In summary, current evidence suggests that botulinum toxin type-A reliably reduces post-stroke spasticity, while RAT can help convert this reduction in tone into meaningful gains in gait and, in selected cases, upper-limb motor performance. When thoughtfully timed and allocated to appropriate candidates, the combined approach appears safe and clinically promising, but larger and more standardized trials are required before it can be recommended as routine care.

### Limitations

This review has important limitations. The total sample size across trials was modest, limiting power to detect differences between treatment strategies. All studies had an overall “some concerns” risk-of-bias rating, mainly due to incomplete reporting and potential selective outcome reporting, so even where patterns appeared consistent—particularly for gait outcomes—they should be interpreted as hypothesis-generating rather than definitive proof of superiority of any specific protocol. Marked heterogeneity in toxin formulations and dosing, robotic devices, training intensity and outcome measures precluded meta-analysis and prevents firm conclusions about optimal protocols. Finally, most participants had chronic stroke, so the generalizability of these findings to acute or subacute phases—when neuroplastic potential may be greater—remains uncertain.

## 4. Conclusions

This systematic review indicates that combining toxin injections with RAT is safe and appears to provide additional functional benefit, particularly for gait, even though it does not consistently enhance spasticity reduction beyond toxin injection plus conventional rehabilitation. Toxin treatment remains the main driver of tone reduction, whereas robotic training may help translate this into improved walking capacity and, in some patients, upper-limb motor performance, especially when started around four weeks after injection. To move from promising data to clear clinical recommendations, future studies should prioritize larger and more standardized randomized trials, rigorous evaluation of timing schedules, and pragmatic, cost-effective models of delivering combined toxin and robotic rehabilitation.

## 5. Materials and Methods

This systematic review was conducted according to the Preferred Reporting Items for Systematic Reviews and Meta-Analyses (PRISMA) guidelines. The review protocol was registered in PROSPERO (CRD42025645965).

### 5.1. Search Strategy

We searched MEDLINE, Embase, Web of Science, the Cochrane Central Register of Controlled Trials (CENTRAL), Scopus and CINAHL from database inception to April 2025 without language restrictions, and screened ClinicalTrials.gov and the WHO International Clinical Trials Registry Platform for ongoing or recently completed trials. We also checked reference lists of relevant articles and conference proceedings. For registered or unpublished trials that appeared eligible, we contacted investigators to clarify recruitment status or request additional data when feasible.

Search strings combined MESH terms related to stroke, spasticity, robot-assisted therapy and botulinum toxin, including both proprietary and non-proprietary names for the main type-A formulations (e.g., Botox/OnabotulinumtoxinA, Dysport/AbobotulinumtoxinA, Xeomin/IncobotulinumtoxinA). The full strategies for each database are reported in Supplementary Table S1.

### 5.2. Eligibility Criteria

We included randomized controlled trials (including cross-over designs) in adults (≥18 years) with a clinical diagnosis of stroke and post-stroke limb spasticity, typically defined by a Modified Ashworth Scale score ≥ 1. Eligible studies evaluated robot-assisted training of the paretic upper or lower limb delivered in combination with botulinum toxin type-A injections, and compared this combined approach with at least one of the following: robot-assisted training alone, toxin injection alone, toxin injection plus conventional therapy, or standard non-robotic rehabilitation. Trials involving non-stroke populations, non-robotic training devices, or no toxin injection were excluded. The primary outcome was change in spasticity measured with validated scales (e.g., Modified Ashworth Scale, Tardieu Scale). Secondary outcomes included motor function (e.g., FMA, Box and Block Test), gait and balance (e.g., 6MWT, TUG, BBS), activities of daily living, quality of life and adverse events. No language or date restrictions were applied during the initial search, although final analysis focused on studies accessible for detailed review. Observational studies and case reports were excluded.

### 5.3. Study Selection and Data Extraction

Two reviewers (S.F., S.S.) independently screened titles and abstracts using the Rayyan platform (https://www.rayyan.ai/, accessed on 1 April 2025), retrieved full texts of potentially eligible studies and assessed them for inclusion; disagreements were resolved by discussion with a third reviewer. The same reviewers extracted data with a standardized form, recording study design, participant characteristics, details of toxin and robotic interventions, comparator treatments, outcome measures and adverse events; funding sources and conflicts of interest were also noted. Any disagreements during this process were resolved through discussion or consultation with a third reviewer (S.A.) if necessary. When required, we contacted corresponding authors to clarify methods or obtain missing data.

We documented the type of robot used, BoNT-A administration protocols, timing of interventions, and duration of treatment. For comparison groups, we recorded details of conventional therapy or control interventions. All outcome measures and their timing were documented, along with complete results and any reported adverse events. We also noted funding sources and potential conflicts of interest.

### 5.4. Risk of Bias

Risk of bias was assessed independently by two reviewers using the Cochrane RoB 2 tool [35] (https://www.riskofbias.info/, accessed on 1 June 2025) across its five specific domains (randomization process, deviations from intended interventions, missing outcome data, measurement of the outcome and selection of the reported result), with disagreements resolved by consensus; an overall judgment (“low risk”, “some concerns” or “high risk”) was assigned to each study based on the ratings across all domains.

### 5.5. Data Synthesis

Because of substantial clinical and methodological heterogeneity in interventions and outcomes, we did not perform a meta-analysis and instead summarized findings using narrative synthesis in line with Cochrane guidance. All stages of the review followed the PRISMA statement, and the protocol remained consistent with the PROSPERO registration (CRD42025645965).

## Figures and Tables

**Table 1:** Overview of Included Studies: Design, Aims, and Participant Characteristics in Trials Combining Robotic Therapy and Botulinum Toxin Type A After Stroke.

Author, Year	Study Aim	Study Design	Participants (N, Characteristics)
Pennati et al. [18] (2015)	Verify how combined short robotic training and chemical neurolysis reduces spasticity and improves function in chronic UL.	Prospective, single-blind RCT, pilot.	N = 15 (Robot only = 8, BoNT-A + Robot = 7). Chronic post-stroke (≥6 mo), severe UL spastic paresis.
Picelli et al. [20] (2016)	Evaluate combined effects of RAT and BoNT-A on spastic equinus foot.	Pilot, single-blind RCT.	N = 22 (Group 1 = 11, Group 2 = 11). Adult outpatients, spastic equinus due to chronic stroke (≥6 mo), FAC ≥ 4.
Erbil et al. [19] (2018)	Investigate combined effects of RAT + PT vs. PT only on balance and gait after BoNT-A.	Prospective RCT.	N = 48 (final N = 29 RAT, N = 14 Control). Chronic stroke (≥6 mo), received BoNT-A for LE spasticity, ambulatory, BBS ≥ 20.
Gandolfi et al. [16] (2019)	Evaluate effects of Robot-assisted UL training on UL spasticity, function, muscle strength, sEMG after BoNT.	Single-blind RCT.	N = 32 (EG = 16, CG = 16). Chronic post-stroke (≥6 mo), UL spastic hemiparesis, MAS (shoulder and elbow) ≤ 3 and ≥1+.
Hung et al. [17] (2022)	Investigate effects of RAT, MT, or AC combined with BoNT-A on motor recovery, spasticity, daily function.	Pilot RCT.	N = 37 (RT = 13, MT = 12, AC = 12). Chronic (≥6 mo) spastic hemiplegic stroke, MAS > 1 (UE), FMA 17–56.
Cotinat et al. [21] (2024)	Compare efficacy of RAT vs. PT on gait after BoNT-A in triceps surae; assess timing.	RCT, cross-over.	N = 33 (15A, 18B). Chronic stroke (≥6 mo), triceps surae spasticity inducing gait impairment.
Shin et al. [15] (2025)	Evaluate combined effects of RAT& BONT-A on UL motor function/spasticity and investigate optimal timing of administration.	Single-blinded, 4-arm RCT, pilot study	N = 42 enrolled, 40 completed. Chronic stroke (≥6 mo), ULS, FMA ≤ 45, MAS elbow flexor ≥ 1+.

Abbreviations: AC, action observation control; BBS, Berg Balance Scale; BoNT-A, botulinum toxin type A; CG, control group; EG, experimental group; FAC, Functional Ambulation Category; FMA, Fugl–Meyer Assessment (Upper Extremity); LE, lower extremity; MAS, Modified Ashworth Scale; MT, mirror therapy; N, sample size; PT, physical therapy; RAT, robot-assisted training; sEMG, surface electromyography; UL/UE, upper limb/upper extremity; ULS, upper limb spasticity.

**Table 2:** Intervention Characteristics, Outcome Measures, and Main Findings of Trials Combining Robotic Therapy and Botulinum Toxin Type A in Post-stroke Spasticity.

First Author, Year	Target Limb BoNT-A Type and Dosage	Robotic Device	Experimental Group	Control Group	Outcome Measures	Key Results on Spasticity	Key Results— Secondary Outcomes
Pennati et al. [18], 2015	Upper Limb Abo Individual dose titration.	ReoGo	BoNT-A + RAT: 2 days/wk, 60 min/sessions– 10 sessions	RAT only: 10 sessions (60 min each, 2–3×/wk) ReoGo UL training.	FMA, B&B Test, MAS, FIM, Euro-Qol, sEMG.	Greater mean decrease in MAS for the BoNT-A + RAT group (change in −0.86) compared to the RAT only group (change of −0.14).	Both groups improved in FMA (RAT only: 8.25; BoNT-A + RAT: 5.29). B&B: RAT only > BoNT-A + RAT. sEMG: Both groups showed improved muscle activation, reduced co-contraction.
Picelli et al. [20], 2016	Lower Limb Abo 250 U per muscle part (Total 750 U).	G-EO System	BoNT-A + ES + RAT: 5 consecutive days, 30 min/day	BoNT-A + Elec. Stim only: No RAT.	MAS, Tardieu scale, 6MWT.	Both groups significantly reduced their MAS scores (*p* < 0.5). No significant between-group difference was found (*p* = 0.852).	6MWT: Group 1 (RAT) > Group 2 (BoNT-A only) for T1-T0 change (diff ~26 m, *p* = 0.045).
Erbil et al. [19], 2018	Lower Limb Abo At least 300 U to plantar flexor group.	RoboGait	BoNT-A + RAT + PT: 3 wks, weekdays. 30 min RAT + 60 min PT per session.	BoNT-A + PT only: 3 wks, weekdays. 90 min PT per session.	MAS, Tardieu Scale, BBS, TUG, RVGA.	Both groups showed significant reductions in MAS. There was no significant between-group difference for MAS (*p* = 0.288 at week 12).	RAT group > Control for TUG, BBS, RVGA change from baseline at Wk6 and Wk12 (*p* < 0.01 for all).
Gandolfi et al. [16], 2019	Upper Limb Ona/Abo/Inco Dosage based on spasticity severity.	Armotion	BoNT-A + RAT: 2 days/wk, 45 min/session (10 min passive mob/stretch + 35 min RAT)–10 sessions	BoNT-A + PT-: 5 wks, 2×/wk, 45 min/session. 10 min passive mob/stretch + 35 min conventional UL exercises.	MAS, FMA, MRC, sEMG (EG only).	Both groups significantly reduced their MAS scores (*p* = 0.008). No significant between-group difference was found (*p* > 0.05).	EG > CG for MRC total and specific movements (*p* < 0.05). sEMG (EG): Biceps activation changes.
Hung et al. [17], 2022	Upper Limb Ona ~306–330 IU total	Manu-Track	BoNT-A + RAT: 3 days/wk, 75 min/session (45 min RAT + 30 min functional practice)- 24 sessions	BoNT-A + Mirror therapy: 8 wks, 3×/wk, 75 min/session. 45 min Mirror Therapy + 30 min functional practice. BoNT-A + PT: 8 wks, 3×/wk, 75 min/session. 45 min conventional task-oriented training + 30 min functional practice.	FMA, MAS, MAL-AOU/QOM, Arm activity level.	All three groups significantly reduced their MAS scores. No significant between-group difference was found (*p* = 0.841).	All 3 groups improved FMA, MAL post-treatment. No between-group difference post-treatment. At 3-mo FU, PT > RT/MT for MAL-QOM (*p* = 0.033). No between-group difference for arm activity level.
Cotinat et al. [21], 2024	Lower Limb Ona 200–204 UI, Inco 200 UI	Lokomat	BoNT-A + RAT then PT: 5 days/wk, 45 min (15 min stretch, 30 min RAT)–10 sessions then 2 wks PT	BoNT-A + PT then RAT: 2 wks PT (5×/wk, 45 min: 15 min stretch, 30 min PT), then 2 wks RAT	6MWT, 10mWT, TUG, BBS, MAS.	Both groups showed significant reductions in MAS. No significant between-group difference in MAS.	RAT > PT for 10 mWT. PT -then- RAT group had better BBS by W8 (*p* = 0.003). W0-W4: RAT > for 6MWT (33 m, *p* = 0.007). W0-W8: RAT -first group maintained 30 m 6MWT advantage (*p* = 0.019).
Shin et al. [15], 2025	Upper Limb Ona Total 300 U	InMotion ARM	BoNT-A + RAT at W0: 5 days/wk, 30 min/sessions from W0–W4–20 sessions	BoNT-A at W0, RAT at W4: BONT-A at W0, then Robot -UL (5 days/wk, 30 min/session–20 sessions) from W4-W8. RAT at W0, BONT-A at W4: RAT (5 days/wk, 30 min/session from W0–W4–20 sessions), then BoNT-A at W4 BoNT-A + RAT at W4: 5 days/wk, 30 min/session from W4–W8–20 sessions	FMA, MAS, Robotic Kinematic Parameters, SIS	W0–W4: groups receiving BoNT-A (either alone or with robot) had a significantly greater spasticity reduction than the groups that did not (*p* < 0.01). W0–W8: no significant difference between the different timing schedules.	W0–W4: No significant Time x Group interactions for FMA. W0-W8: Group BONT-A at W0, RAT at W4 showed the most substantial and significant improvement in FMA and kinematic parameters compared to the reference group (*p* = 0.042).

Abbreviations: 6MWT, 6-Minute Walk Test; 10mWT, 10-Meter Walk Test; B&B, Box and Block Test; BBS, Berg Balance Scale; BoNT-A, botulinum toxin type AEG, experimental group; ES, electrical stimulation; FIM, Functional Independence Measure; FMA, Fugl–Meyer Assessment; FU, follow-up; Inco, incobotulinumtoxinA; MAS, Modified Ashworth Scale; MRC, Medical Research Council Scale; MT, mirror therapy; Ona, onabotulinumtoxinA; PT, physical therapy; RAT, robot-assisted training; RVGA, Rivermead Visual Gait Assessment; sEMG, surface electromyography; SIS, Stroke Impact Scale; TUG, Timed Up and Go Test; UL/UE, upper limb/upper extremity; W, week.

## Data Availability

No new data were created or analyzed in this study.

## Supplementary Materials

The following supporting information can be downloaded at: https://www.mdpi.com/article/10.3390/toxins17120569/s1, Table S1: search strategy.
