# Supplementary material for: Botulinum Toxin Combined with Robot-Assisted Therapy for Post-Stroke Spasticity: A Systematic Review
Source: Toxins (Basel). 2025 Nov 25;17(12):569. doi: 10.3390/toxins17120569 (PMC12737565; doi:10.3390/toxins17120569)
Supplement: Supplementary file 1 [file toxins-17-00569-s001.zip › toxins-3978717-supplementary.pdf]

Supplementary Materials: Botulinum Toxin Combined with Robot-Assisted Therapy for Post-Stroke Spasticity: A Systematic Review

Author information removed for double blind peer review

Table S1. Search Strategy

PUB-MED+A1:B7	(("Botulinum Toxins, Type A"[Mesh] OR "botulinum toxin" OR "BoNT-A" OR "BTX-A" OR "Botox" OR "Dysport" OR "Xeomin")<br>AND<br>("Robotics"[Mesh] OR "robot-assisted" OR "robotic therapy" OR "robotic rehabilitation" OR "exoskeleton" OR "robotic device" OR "assistive device")<br>AND<br>("Muscle Spasticity"[Mesh] OR "spasticity" OR "muscle hypertonia" OR "spastic*")<br>AND<br>("Stroke"[Mesh] OR "stroke" OR "post-stroke" OR "chronic stroke")<br>AND<br>("Randomized Controlled Trial"[Publication Type] OR "randomized controlled trial" OR "RCT" OR "clinical trial"))<br>NOT<br>("Animals"[Mesh] NOT "Humans"[Mesh])	7
Scopus	(TITLE-ABS-KEY (("botulinum toxin type a" OR "bont-a" OR "btx-a" OR botox OR dysport OR xeomin OR "botulinum toxin") AND (robot* OR "robotic therapy" OR "robot-assisted" OR exoskeleton OR "robotic device" OR "assistive device") AND (spasticity OR "muscle hypertonia" OR spastic*) AND (stroke OR "post-stroke" OR "chronic stroke") AND ("randomized controlled trial" OR rct OR "clinical trial"))) ANDNOT TITLE-ABS-KEY (animal* OR rat OR mice OR murine) AND (LIMIT-TO (DOCTYPE , "ar") OR LIMIT-TO (DOCTYPE , "ct"))	13
Web of science	(TS=(<br>("botulinum toxin type a" OR "bont-a" OR "btx-a" OR botox OR dysport OR xeomin OR "botulinum toxin")<br>AND<br>(robot* OR "robotic therapy" OR "robot-assisted" OR exoskeleton OR "robotic device" OR "assistive device")<br>AND<br>(spasticity OR "muscle hypertonia" OR spastic*)<br>AND<br>(stroke OR "post-stroke" OR "chronic stroke")<br>AND<br>("randomized controlled trial" OR RCT OR "clinical trial")))<br>NOT TS=(animal* OR rat OR mice OR murine)	10

Cochrane library	(("botulinum toxin type a" OR "bont-a" OR "btx-a" OR botox OR dysport OR xeomin OR "botulinum toxin")<br>AND<br>(robot* OR "robotic therapy" OR "robot-assisted" OR exoskeleton OR "robotic device" OR "assistive device")<br>AND<br>(spasticity OR "muscle hypertonia" OR spastic*)<br>AND<br>(stroke OR "post-stroke" OR "chronic stroke")<br>AND<br>("randomized controlled trial" OR RCT OR "clinical trial"))<br>NOT (animal* OR rat OR mice OR murine)	19
Embase	('botulinum toxin a'/exp OR 'botulinum toxin' OR 'bont-a' OR 'btx-a' OR 'botox' OR 'dysport' OR 'xeomin') AND ('robotics'/exp OR 'robot-assisted' OR 'robotic therapy' OR 'robotic rehabilitation' OR 'exoskeleton' OR 'robotic device' OR 'assistive device') AND ('stroke'/exp OR 'post-stroke' OR 'chronic stroke') AND ('randomized controlled trial'/exp OR 'clinical trial'/exp OR 'rct') NOT ('animal'/exp NOT 'human'/exp)	15
PEDRo	Robot Botulinum Toxin stroke	1
MEDLINE (OVID)	((exp "Botulinum Toxins, Type A"/ OR "botulinum toxin".tw. OR BoNT-A.tw. OR BTX-A.tw. OR Botox.tw. OR Dysport.tw. OR Xeomin.tw.)<br>AND<br>(exp Robotics/ OR "robot-assisted".tw. OR "robotic therapy".tw. OR "robotic rehabilitation".tw. OR exoskeleton.tw. OR "robotic device".tw. OR "assistive device".tw.)<br>AND<br>(exp Stroke/ OR stroke.tw. OR post-stroke.tw. OR "chronic stroke".tw.)<br>AND<br>("Randomized Controlled Trial".pt. OR "randomized controlled trial".tw. OR RCT.tw. OR "clinical trial".tw.))<br>NOT (exp Animals/ NOT exp Humans/)	7
Clinicaltrials.gov	Condition/disease<br>Stroke<br>Other terms<br>spasticity<br>Intervention/treatment<br>Botulinum Toxin Type A and Robotic	7
WHO ICTRP	Stroke AND Spasticity AND Robotics AND Botulinum Toxin	1
